# TALASUR trial: a single arm phase II trial assessing efficacy and safety of TALazoparib and Avelumab as maintenance therapy in platinum-Sensitive metastatic or locally advanced URothelial carcinoma

**DOI:** 10.1186/s12885-022-10216-z

**Published:** 2022-11-24

**Authors:** Elodie Coquan, Bénédicte Clarisse, Justine Lequesne, Pierre-Emmanuel Brachet, Zoé Nevière, Emeline Meriaux, Isabelle Bonnet, Marie Castera, Nicolas Goardon, Jeremy Boutrois, Romain Travers, Florence Joly, Jean-Michel Grellard, Antoine Thiery-Vuillemin

**Affiliations:** 1 Medical Oncology Department, Centre François Baclesse, 3 Avenue du Général Harris, 14076 Caen Cedex 05, France; 2 Clinical Research Department Centre François Baclesse, 14076 Caen, France; 3 Genetic and Oncology Biology Department, Centre François Baclesse, 14076 Caen, France; 4 Northwest Data Center (CTD-CNO), Centre François Baclesse, 14076 Caen, France; 5 Medical Oncology Department, CHRU Besançon Hôpital Jean Minjoz, 25030 Besançon, France

**Keywords:** Urothelial carcinoma, PARP inhibitors, Immune checkpoint inhibitors, Anti–PD-L1, Maintenance treatment

## Abstract

**Background:**

Urothelial carcinoma (UC) is the ninth most commonly diagnosed cancer worldwide, with a 3.8/1 male to female ratio. Platinum-based chemotherapy is the first line standard of care for fit patients with advanced UC. However, despite a response rate (RR) for approximately half of patients receiving standard chemotherapy, durable responses are rare (median progression-free progression (PFS) around 8 months). Recently, immune checkpoint inhibitors (ICI) have emerged as new therapeutic options. Among them, Avelumab, an anti-PD-L1 antibody, was assessed in maintenance treatment, demonstrating an overall survival improvement in the JAVELIN Bladder-100 phase III trial. These findings led to its approval as first line maintenance therapy for patients with locally advanced or metastatic UC who have not progressed on prior platinum-containing chemotherapy. However, disease progression as best response was noticed for 37% of patients under Avelumab as maintenance treatment.

UC has targetable genomic alterations, including DNA damage repair (DDR) alterations. DDR deficiency is known to major sensitivity to both platinum-based chemotherapy and PD-1/PD-L1 blockade and the combination of ICI and PARP inhibitors showed promising results. It therefore warrants to assess the interest of combining ICI plus PARP inhibitors as maintenance treatment in UC patients.

**Methods:**

The TALASUR trial is a single-arm multicenter phase 2 study aiming to assess the antitumor activity of the combination of Avelumab with Talazoparib among patients with locally advanced/metastatic UC in maintenance therapy after platinum-based chemotherapy.

The primary objective is to determine the efficacy of the combination, assessed through PFS. Secondary objectives are as follows: safety profile of the association, objective response, duration of tumoral response, disease control rate, time to subsequent therapy, quality of life. A blood and tumor collections will be also constituted.

Patient will receive the combination therapy of daily oral Talazoparib (1 mg/day) and intra-venous Avelumab 800 mg on days 1 and 15, in a 28-day cycle. Fifty patients will be enrolled.

**Discussion:**

Talazoparib with Avelumab combination may have additive activity when administrated jointly. We hypothesize that combination will increase the antitumor activity in UC first line maintenance setting with an acceptable safety profile.

**Trial registration:**

NCT04678362, registered December 21, 2020. Protocol version: Version 1.3 dated from 2020 09 11.

## Background

### Metastatic urothelial carcinoma management

Significant improvements were made in the management of metastatic urothelial carcinoma (mUC) with immune checkpoint inhibitors (ICI). In first line setting, for patients who are deemed ineligible for platinum-based therapy but whose tumor expresses PD-L1, 2 checkpoint inhibitors (Pembrolizumab, Atezolizumab) are currently approved. After platinum-based chemotherapy failure, five molecules anti-PD-1 or PD-L1 antibodies (Pembrolizumab, Atezolizumab, Avelumab, Nivolumab and Durvalumab) have obtained FDA/EMA approval [1, 2]. More recently, the Javelin Bladder 100 phase III trial assessing Avelumab, an anti-PD-L1 antibody, as switch maintenance therapy in mUC patients who achieved stable disease or response after 4–6 cycles of first line platinum-based chemotherapy. The study demonstrated significantly prolonged overall survival (OS) with maintenance Avelumab + Best Supportive Care (BSC) vs BSC alone (median OS = 21.4 versus 14.3 months, *p* < 0.001) as well as progression-free survival (PFS) (median PFS 3.7, versus 2.0 months, *p* < 0.001) [3]. The objective response rate (ORR) was 9.7% of patients in the Avelumab + BSC arm versus 1.4% in the BSC alone arm. The safety profile was similar to that of other ICI. Despite these encouraging results showing long term durable ORR, 37% of patients had disease progression as best response. There is thus a need to find alternative/combination approaches for the treatment of mUC.

### The emerging role of DNA damage repair and PARP inhibition in UC

The characterization of the genomic landscape of bladder UC has identified targetable genomic alterations, including a high number of loss of function mutations in DNA damage repair (DDR) genes gathered in the concept of “BRCAness phenotype” [4, 5]. The Cancer Genome Atlas analysis of bladder UC found 34% of BRCAness phenotype including *CHEK1/2*, *RAD51*, *BRCA1/2*, *ATM*, *ATR*, *MDC1*, and *FANCF* gene alterations [4]. Necchi et al. underwent a comprehensive genomic profiling of 479 upper tract UC and 1 984 bladder UC: they found 4.6 and 4.9% of specific *BRCA1/2* alterations, respectively [5]. Studies that evaluated the frequency of germline pathogenic variants in the DDR genes found alterations in 10% of cases [6, 7].

The “BRCAness phenotype” has been shown to confer sensitivity to the Poly ADP-Ribose Polymerase (PARP) inhibitors. Several PARP inhibitors (PARPi) are nowadays approved in various tumor types (breast, prostate, pancreas and ovarian carcinoma) [8]. For some tumor types, the benefit is not restricted to BRCA mutation (e.g. ovarian, breast or prostate cancer). PARPi have also demonstrated efficacy in unselected-biomarkers ovarian carcinoma, in maintenance setting after response to platinum-based chemotherapy.

The prevalence of these somatic mutations in homologous recombination (HR) pathway as well as their association with improved platinum sensitivity suggests PARP inhibition to be a rational target for the treatment of mUC, but the biomarker selection remains unclear.

PARPi were assessed in monotherapy or in association in UC patients, molecularly selected or not. The ATLAS trial failed to demonstrate the efficacy of Rucaparib, a PARPi as single agent treatment in patients with previously treated locally advanced/unresectable or metastatic UC, in a biomarker-unselected population [9]. Similarly, the Meet-Uro12 trial, a randomised phase II study, failed to demonstrate the efficacy of niraparib monotherapy plus BSC versus BSC alone as maintenance treatment for patients with unresectable or metastatic biomarker-unselected UC whose disease did not progress after first line platinum-based chemotherapy [10, 11]. In this study, BRCAness phenotype was retrospectively evaluated: HRR alterations were found in 44.7% of patients, illustrating platinum sensitivity of mUC with DDR deficiency. In contrast, the Atlantis trial is a biomarker-directed, multi-arm phase II study, in maintenance setting for mUC after platinum-based chemotherapy response or stable disease [12, 13]. Results from the Atlantis rucaparib arm in DDR-selected mUC were recently presented: maintenance rucaparib was associated with promising benefit as compared to placebo (median PFS 35 versus 15 weeks respectively, HR 0.53, 80% CI 0.30–092; *p* = 0.07). Similarly, the BISCAY trial is an open-label, biomarker-directed, multi-arm phase Ib study in mUC after platinum-based chemotherapy failure [14]. DDR deficiency was found in 14% of tumors and interestingly, 54% of these tumors were considered tumor mutation burden (TMB) high. The combination of olaparib, a PARPi and durvalumab, an anti-PD-L1 inhibitor, demonstrated modest but interesting clinical benefit, with an ORR of 35.7% in this biomarker-selected population.

Maintaining a tumoral response and prolonging PFS are clinically meaningful purposes for clinical trials exploring novel agents in advanced UC. The objectives of a maintenance strategy are to increase and/or prolong the benefit of chemotherapy using drugs which safety profile allowing their long term use.

### Rationale for combining Talazoparib and Avelumab

PARPi and ICI are good candidates thanks to their acceptable safety profile, and synergistic activity. They are already approved in this maintenance strategy. Preclinical and clinical data suggest that PARPi may have additive activity when administrated in combination with ICI, through different mechanisms, independently of the DNA damage repair status [15, 16]: PARPi-mediated unrepaired DNA damages indeed modulate the tumor immune microenvironment which might promote responsiveness to ICIs.

The Neodurvarib trial assessed Durvalumab in combination with Olaparib in UC neo-adjuvant setting. It highlighted promising results, with complete pathological response for 44.5% of patients and acceptable toxicity [17].

Talazoparib is an orally bioavailable potent inhibitor of PARP [18]. It selectively binds to PARP and prevents PARP-mediated DNA repair of single strand DNA breaks via the base-excision repair pathway. This enhances the accumulation of DNA strand breaks, promotes genomic instability and eventually leads to apoptosis. In a first-in-man phase 1 study, Talazoparib showed clinical activity in various solid tumors. It is already approved for the treatment of metastatic breast cancer patients with an inherited BRCA mutation [19, 20].

The phase Ib/II trial assessing the association of Avelumab and Talazoparib (Javelin PARP Medley trial—NCT03330405) in locally advanced or metastatic solid tumors, is ongoing [21]. Results from the phase Ib step indicate an acceptable safety profile of the combination. The RP2D (recommended phase 2 dose) was Avelumab 800 mg per intravenous route every two weeks (Q2W) and Talazoparib 1 mg per os daily given (0.75 mg daily in case of moderate renal impairment). Results from phase II biomarker-selected advanced breast cancer cohorts (22 and 23 patients) were also presented. Beyond the acceptable safety profile, the combination demonstrated meaningful antitumor activity, with 18% and 30% ORR according to the cohorts and responses generally durable. The 3-arm phase III trial Javelin Ovarian PARP100 was designed to evaluate the efficacy and safety of avelumab in combination with chemotherapy followed by maintenance therapy of avelumab plus talazoparib in treatment-naïve patients with locally advanced or metastatic ovarian cancer. The trial was stopped prematurely for futility (insufficient efficacy)of avelumab monotherapy in this setting but did not showed safety concerns about the association [22].

### Purpose

We propose to investigate the efficacy and the safety of the association Talazoparib and Avelumab as maintenance therapy in platinum-sensitive metastatic or locally advanced UC. Patients will be selected according to platinum sensitivity, to allow excluding poor prognosis tumors and potentially enriching the proportion of patients whose tumor harbours alterations in genes involved in DNA damage repair. Predictive interest of DDR biomarkers will be assessed as part of ancillary analyses.

## Methods / design

We propose a one-arm single-stage multicenter phase II study aiming to assess the interest of combining Talazoparib and Avelumab as maintenance therapy in platinum-sensitive metastatic or locally advanced UC. The TALASUR protocol and this manuscript have been written in accordance with standard protocol items, namely recommendations for interventional trials (SPIRIT).

### Primary outcome

The primary objective of this phase II study is to determine the efficacy of a maintenance treatment combining Talazoparib with Avelumab in patients with locally advanced/metastatic UC with a stable disease or objective response to a platinum-based chemotherapy.

Efficacy will be measured by the PFS defined as the time from initiation of Talazoparib plus Avelumab treatment to the first documented disease progression per RECIST 1.1 criteria (Response Evaluation Criteria in solid Tumors) based on investigator’s assessment, or death due to any cause, whichever occurs first.

### Secondary outcomes

The secondary objectives are to evaluate:

- ▪The safety profile of Talazoparib plus Avelumab according to NCI CTCAE v5.0 criteria
- ▪The efficacy of the combination, for whole study population, and according to the response to the initial chemotherapy, through:
  • Overall Survival, defined as the time from Talazoparib + Avelumab initiation to death whatever cause
  • ORR, defined as the proportion of patients with partial or complete response, according to RECIST 1.1 criteria
  • ORR, defined as the proportion of patients with partial or complete response, according to RECIST 1.1 criteria
  • Duration of tumoral response, defined as the time elapsed between first date of objective response (complete or partial response) and date of first documented disease progression according to RECIST 1.1 criteria or date of death, due to any cause, whichever occurs first
  • Disease control rate, defined as the proportion of patients with stable disease, partial or complete response, according to RECIST 1.1 criteria
  • Duration of treatment by Talazoparib plus Avelumab taken together and separately defined as the time elapsed between the first dose of treatment and the date of permanent discontinuation of Talazoparib and/or Avelumab regardless of cause (Patients still on treatment with any study drug at the time of database lock will be censored).
  • Time to subsequent therapy (TST), defined as the time elapsed between the date of initiation of Talazoparib plus Avelumab and the date of initiation of subsequent systemic therapy, respectively (Patients who did not receive any subsequent treatment or who have died prior to receiving subsequent treatment will be censored).
- ▪To assess Quality of life (QoL) of patients under Talazoparib plus Avelumab treatment with:
  •Scores of quality of life assessed through the French version of the self-administered standardized questionnaire EORTC QLQ-C30 and EuroQoL EQ-5D.
  •Survival without deterioration in QoL as assessed using the QLQ-C30 questionnaire.
- ▪To constitute a blood and tumor banking for ancillary biological explorations.

These translational researches may include the determination of the patients’ and disease’s characteristics of tumors (PD-L1 status, microsatellite instability (MMR), tumor mutation burden (TMB), specific molecular alterations of the homologous recombination pathway from genetic material derived from tumor tissue or circulating tumoral DNA). The relationship between clinical and molecular (genomic, metabolic, and/or proteomic) markers and the safety profile, as well as the efficacy of the combination Avelumab plus Talazoparib may also be considered.

### Study population

Eligibility criteria are precised in Table 1. The TALASUR study addresses patients with locally advanced/metastatic UC with a stable disease or objective response to a platinum-based chemotherapy.

**Table 1:** Study eligibility criteria

Inclusion criteria	- Patient ≥ 18 years at the day of consenting to the study - Provision of informed consent prior to any study specific procedures - Histologically confirmed diagnosis of urothelial carcinoma of the renal pelvis, ureter (upper urinary tract), bladder or urethra. Both transitional cell and mixed transitional/non-transitional cell histologies are allowed, but transitional cell carcinoma must be the predominant histology - Documented Stage IV disease (T4b, N0, M0; any T, N1–N3, M0; any T, any N, M1) not candidate to a curative treatment with surgery or radiotherapy at the start of first-line platinum-based chemotherapy - Patient must have completed prior to inclusion a platinum-based (cisplatin or carboplatin) polychemotherapy for at least 4 cycles of chemotherapy (until 6 cycles maximum) and have a stable disease or a partial response (PR) or a complete response (CR) from the chemotherapy according to RECIST 1.1 criteria ◦ A minimum dose of 55 mg/m^2^ of cisplatin is required in order to count for 1 cycle ◦ A minimum dose of carboplatin AUC 4.5 is required in order to count for 1 cycle Disease response will be established locally by the investigator by examining pre and post-chemotherapy radiological assessments (CT/MRI) ◦ Neoadjuvant or adjuvant chemotherapy is allowed (with a delay of at least 12 months between the last dose of neoadjuvant or adjuvant chemotherapy and relapse) - Patient must be enrolled within 8 weeks after the last dose of chemotherapy - Eastern Cooperative Oncology Group (ECOG) performance status ≤ 2; - Normal organ and bone marrow function measured within 28 days prior to administration of study treatment as defined below: ◦ Hemoglobin ≥ 10.0 g/dL (patient may have been transfused before inclusion) ◦ Absolute neutrophil count (ANC) ≥ 1.5 × 109/L ◦ Platelet count ≥ 100 G/l ◦ Total bilirubin ≤ 1.5 × institutional upper limit of normal (ULN) ◦ Aspartate aminotransferase (AST) (Serum Glutamic Oxaloacetic Transaminase (SGOT)) / Alanine aminotransferase (ALT) (Serum Glutamic Pyruvate Transaminase (SGPT)) ≤ 2.5 × ULN unless liver metastases are present in which case they must be ≤ 5 × ULN ◦ Patient must have creatinine clearance estimated using the CKD equation of ≥ 40 mL/min - Able to swallow and retain oral drug - Life expectancy > 12 weeks - Serum pregnancy test (for females of childbearing potential) negative at screening - Male patient able to father children and female patient of childbearing potential and at risk for pregnancy must agree to use 2 highly effective methods of contraception throughout the study and for at least 60 days after the last dose of treatments - Patient affiliated to a French Social Security System or a beneficiary of such a system - Patient willing and able to comply with the protocol for the duration of the study including undergoing treatment and scheduled visits and examinations - Optional: provision of a recent formalin-fixed, paraffin-embedded (FFPE) tumor tissue block (or subsection thereof) from the most recent primary or metastatic tumor biopsy
Non-inclusion criteria	- Patient who has never received chemotherapy with a platinum salt (cisplatin or carboplatin) for advanced/metastatic urothelial carcinoma - Patient who has previously received more than one line of chemotherapy for advanced/metastatic urothelial carcinoma - Patient whose disease has progressed according to RECIST v1.1 criteria after the first line platinum-based chemotherapy for urothelial carcinoma. The cancer must not be in the progression phase at inclusion - Patient with known central nervous system metastases and/or carcinomatous meningitis - Other malignancy within the last 3 years except: adequately treated non-melanoma, skin cancer curatively treated, in situ cancer of the cervix, ductal carcinoma in situ (DCIS), localized prostate carcinoma without PSA relapse - Patient with myelodysplastic syndrome/acute myeloid leukemia history or with features suggestive of MDS/AML - Active autoimmune disease that might deteriorate when receiving an immuno-stimulatory agent. Patient with diabetes type I, vitiligo, psoriasis, or hypo- or hyperthyroid diseases not requiring immunosuppressive treatment are eligible. Replacement therapy (e.g., thyroxine, insulin, or physiologic corticosteroid replacement therapy for adrenal or pituitary insufficiency, etc.) are allowed. Current treatment with an immunosuppressant medicinal product or treatment within 7 days prior to inclusion, EXCEPT: ◦ Intra-nasal, inhaled or local steroids or local steroid injections (such as intra-articular injections) ◦ Systemic corticosteroids at physiological doses of ≤ 10 mg/day of prednisone or equivalent ◦ Steroids as premedication for hypersensitivity reactions (such as CT scan premedication) - Major surgery within 4 weeks or major radiotherapy within to starting experimental treatment. Previous palliative radiotherapy (≤ 10 fractions) for metastatic lesions is permitted provided that this has been completed at least one week prior to starting Talazoparib and Avelumab - Active viral infection (HIV, Hepatitis B/C) or known history of positive test for HIV - Any previous treatment with PARP inhibitor or any immunotherapy (e.g. anti-CTLA-4 or anti-PDL1/ PD1) - Concomitant treatment with any drug on the prohibited medication list such as live vaccines, concomitant use of strong P-gp inhibitors (cf section “Prohibited concomitant treatments”) or systemic corticoids at dose > 10 mg/day prednisone or equivalent. Live vaccines administered more than 30 days before study entry are permitted - Clinically significant (e.g. active) cardiovascular disease cerebral vascular accident/stroke in the 3 months prior to enrollment: myocardial infarction, severe/unstable angina, symptomatic congestive heart failure (≥ New York Heart Association Classification Class II), serious cardiac arrhythmia requiring medication, uncontrolled high blood pressure, cerebrovascular accident, transient ischaemic attack - Patient considered at poor medical risk due to a serious, uncontrolled medical disorder, non-malignant systemic disease or active, uncontrolled infection - Pulmonary embolism or deep vein thrombosis within 3 months prior to inclusion (unless if stable, asymptomatic and treated with a low molecular heparin for at least 10 days prior to starting Talazoparib + Avelumab) - Pregnant or lactating woman; - Participation in another interventional study with a systemic anti-cancer treatment within 4 weeks prior to inclusion. Inclusion in observational or interventional studies not involving a health product is permitted. Patient with telephone follow up of toxicities and simple laboratory monitoring or other questionnaires alone may be included - Patient unable to swallow orally administered medication and patients with gastrointestinal disorders likely to interfere with absorption of the study medication; - Previous organ transplant including stem cell allotransplantation or double umbilical cord blood transplantation - Patient with a known hypersensitivity to Talazoparib and Avelumab or any of the excipients of the product - People who are vulnerable under the law (minors, adults under legal protection, people deprived of their freedom) - Other persisting toxicities related to previous anticancer treatments: “Persisting toxicity related to prior therapy (NCI CTCAE Grade > 1); however, alopecia, sensory neuropathy Grade ≤ 2, or other Grade ≤ 2 not constituting a safety risk based on investigator’s judgment are acceptable.”

### Study sites

The list of study sites is indicated on https://clinicaltrials.gov/ct2/show/NCT04678362. The participation of 12 French Centres highly involved in the GETUG intergroup is planned (Table 2).

**Table 2:** Participating centers to the TALASUR trial

INVESTIGATORS	PARTICIPATING FRENCH CENTRES
**Principal investigator:** Dr Elodie COQUAN **Co-investigators:** Prof Florence JOLY Dr Emeline MERIAUX Dr Pierre-Emmanuel BRACHET Dr Emmanuel MEYER Dr Isabelle BONNET	**Centre François Baclesse, CAEN**
**Principal investigator:** Dr Ahmed KHALIL **Co-investigators:** Dr Marc-Antoine BENDERRA Dr Djamel GHEBRIOU	**Hôpital Tenon, PARIS**
**Principal investigator:** Dr Antoine THIERY VUILLEMIN **Co-investigators:** Dr Tristan MAURINA Dr Guillaume MOUILLET Dr Fabien CALCAGNO Dr Hamadi ALMOTLAK Dr Ulrich STEIN Dr Thierry NGUYEN	**CHU Jean Minjoz, BESANCON**
**Principal investigator:** Dr Aude FLECHON **Co-investigators:** Dr Helen BOYLE Dr Kathleen DEKEISTER Dr Armelle VINCENEUX	**Centre Léon Bérard, LYON**
**Principal investigator:** Dr Hakim MAHAMMEDI **Co-investigators:** Dr isabelle VAN PRAAGH Dr Camille POIRRIER	**Centre Jean Perrin, CLERMONT-FERRAND**
**Principal investigator:** Dr Camille SERRATE **Co-investigators:** Dr Antoine ANGELERGUES	**Croix Saint-Simon Diaconesses, PARIS**
**Principal investigator:** Dr Sylvain LADOIRE **Co-investigators:** Dr Coureche KADERBHAI Dr Sylvie ZANETTA	**Centre George-François Leclerc, DIJON**
**Principal investigator:** Dr Elouen BOUGHALEM **Co-investigators:** Dr Frederic ROLLAND Dr Emmanuelle BOMPAS Dr Damien VANSTEENE Dr Cyriac BLONZ	**Institut de Cancérologie de l’Ouest, NANTES**
**Principal investigator:** Dr Elouen BOUGHALEM **Co-investigators:** Dr Remy DELVA Dr Sophie ABADIE-LACOURTOISIE Dr Manon DE VRIES Dr Frédéric BIGOT Dr Victor SIMMET	**Institut de Cancérologie de l’Ouest, ANGERS**
**Principal investigator:** Dr Damien POUESSEL **Co-investigators:** Dr Christine CHEVREAU Dr Iphigénie KORAKIS Dr Sarah BETRIAN Dr Carlos GOMEZ-ROCA Dr Loic MOUREY Dr Valentin THIBAUD Dr Marion ROLLAND	**Institut Universitaire de Cancérologie de Toulouse, TOULOUSE**
**Principal investigator:** Dr Delphine BORCHIELLINI **Co-investigators:** Dr Nicolas MARTIN Dr Rémy LARGILLIER Dr Caroline BAILLEUX Dr Esma SAADA BOUZID Dr Jérôme BARRIERE	**Centre Antoine Lacassagne, NICE**
**Principal investigator:** Dr Laurence CROUZET **Co-investigators:** Dr Brigitte LAGUERRE Dr Héloïse BOURIEN Dr Romain KOKORIAN Dr Lea MUZELLEC	**Centre Eugène Marquis, RENNES**
**Principal investigator:** Dr Friederike SCHLUMANN **Co-investigator:** Dr Benjamin AUBERGER	**CHU, BREST**

### Study treatments and procedures

**Fig. 1:**
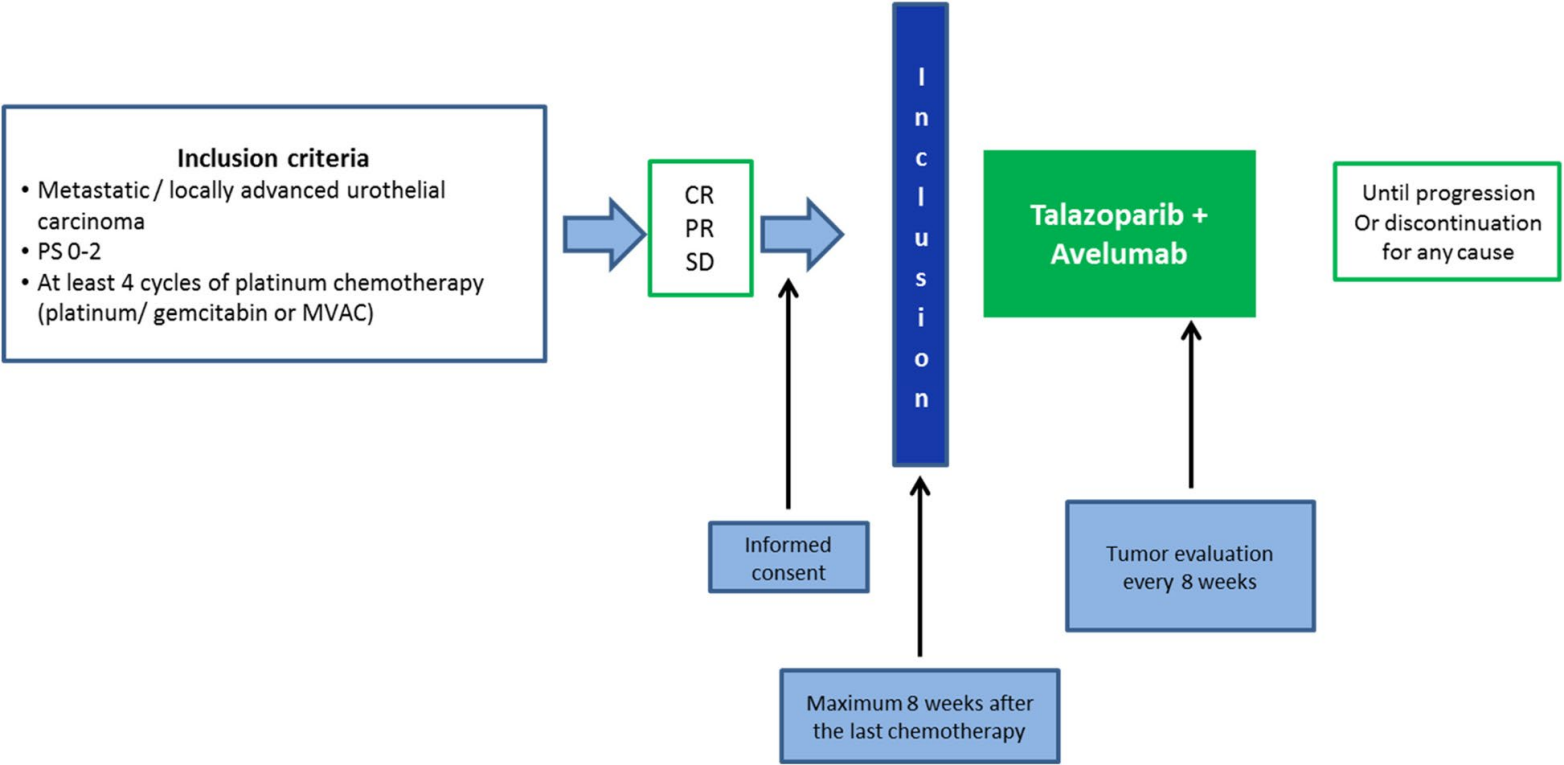
TALASUR study schedule

The study schedule is resumed in Fig. 1 and an overview of study assessments and procedures is presented in Table 3.

**Table 3:** Overview of study assessments of the TALASUR trial

	**Screening** (within 14 days prior C1D1)	**During treatment** **(1 cycle = 28 days)**		**End of treatment** *(within 4 weeks after last dose)*	**Follow-up after the end of treatment**	**Overall survival after disease progression**
		**Every cycle**				
		**D1**	**D15**			
**Informed Consent** should be done before any study procedures						
**Informed consent**	✔					
Inclusion/Exclusion Criteria	✔					
Demographics - Medical/surgical history and prior treatment for urothelial carcinoma	✔					
Prior and Concomitant Medication Review	✔	✔	✔	✔		
**Trial treatment administration**		✔	✔			
Complete physical, weight, height (only at baseline), ECOG, vital signs	✔^**a**^	✔	✔	✔	✔^**i**^	
**Adverse Events (AEs) collection**		✔	✔	✔	✔^**i**^	
**CBC, platelets, hemoglobin**	✔^**a**^	✔	✔	✔		
**Blood biochemistry**	✔^**a,b**^	✔^**b**^ *(cycle 1: within 7 days prior C1D1)*	✔^**g**^	✔^**b**^		
**Coagulation**	✔^**a,c**^	✔*(if clinically indicated)*				
**Thyroid-function testing:** thyroid-stimulating hormone [TSH], free T4	✔^**a**^	✔^**h**^		✔		
HIV, HBV and HCV serology	✔^**a,d**^					
Serum or urine pregnancy test (if applicable)	✔^**a**^	✔				
**Urinalysis**	✔^**a,e**^	✔*(if clinically indicated*^**e**^		✔*(if clinically indicated)*^**e**^		
**ECG**	✔*(within 28 days prior C1D1)*	✔*(if clinically indicated)*				
**Tumor imaging**^**f**^	✔*(within 28 days prior C1D1)*	✔*(Every 8 weeks)*		✔	✔^**j**^	
**Quality of life** (QLQ-C30 and EQ-5D)	✔^**a**^	✔*(Every 8 weeks)*		✔		
**Patient diary**		✔				
**Survival status**						✔^**k**^
**BLOOD and TUMOR BANKING for further ancillary biological studies for patients having given their specific informed consent**						
**Blood samples**	✔^**l**^					
**Fixed primitive tumor sample (or 20 slides)**	✔					

^a^Within 7 days before inclusion

^b^Serum biochemistry at inclusion, Day 1 of each cycle and end of treatment: Sodium, potassium, chloride, corrected calcium, total bilirubin, creatinine, glucose, albumin, total protein, GGT, ALT, AST, alkaline phosphatase, LDH, lipase, magnesium

^c^INR and aPTT or PTT

^d^Human Immunodeficiency Virus, Hepatitis B surface antigen (HBsAg), antibodies against HBsAg, total hepatitis B core antibody (HBcAb); hepatitis C virus antibody (anti-HCV)

^e^If ≥ 2 + , collect 24-h urine collection

^f^Tumor assessments will include all known or suspected disease sites. Imaging includes chest, abdomen, and pelvis computed tomography (CT) or magnetic resonance imaging (MRI) scans if CT is contra-indicated. Bone scans or brain MRI scans may be realised if clinically indicated by clinical investigator. Bone scans will only be repeated during study as clinically indicated. The CT and MRI scans should be performed with contrast agents unless contraindicated for medical reasons. The same imaging technique used to characterize each identified and reported lesion at baseline will be employed in the following tumor assessments

^g^Serum biochemistry at Day 15 of each cycle: Sodium, potassium, chloride, corrected calcium, total bilirubin, creatinine, glucose, albumin, total protein, GGT, ALT, AST, alkaline phosphatase, LDH

^h^Only on Day 1 of cycle 2, 3 and every 2 cycles

^i^At one and 3 months after the last dose

^j^For patients who have stopped the treatment for another reason than disease progression: tumoral evaluation every 8 weeks up to disease progression

^k^Every 3 months

^l^Blood samples at baseline

Eligible patients must be enrolled by medical oncologists within 8 weeks after the last dose of chemotherapy and start the combined treatment within 28 days after inclusion. Patients will receive a treatment by Talazoparib plus Avelumab as follows:

- Talazoparib will be administered at the daily dose of 1 mg given orally in a 28-day cycle.
  In case of mild renal impairment (creatinine clearance between 30-59 mL/min) at inclusion, patients will receive the daily dose of 0.75 mg.
- Avelumab will be administered by intravenous (I.V.) route over 60 min at the dose of 800 mg on D1 and D15, in a 28-day cycle.

For Avelumab treatment, patients have to be premedicated with an antihistamine and with paracetamol 30 to 60 min prior to the first 4 infusions. If the fourth infusion is completed without an infusion-related reaction, premedication for subsequent doses should be administered at the discretion of the physician. No premedication is required for Talazoparib.

Any toxicity observed during the course of the study could be managed by discontinuation of Talazoparib or dose reduction based on severity and clinical presentation. A maximum of three dose reductions are allowed for Talazoparib: it is recommended to first reduce to 0.75 mg daily, then to 0.5 mg daily, and lastly to 0.25 mg.daily. As for Avelumab, there is no dose adaptation: dosing delay or discontinuation may be required based on individual safety and tolerability.

Concomitant treatments must be prescribed in accordance with the Avelumab and Talazoparib and collected in the e-CRF.

The combined treatment will be continued until disease progression, unacceptable toxicity or discontinuation for any cause (patient or investigator consideration).

The patient’s participation in the study will be stopped once the treatment with both study drugs will be discontinued regardless of cause. A period of 8 weeks with no doses of Avelumab and/or Talazoparib is deemed to be treatment discontinuation and withdrawal of the patient from the study. Subsequent care will be given outside of the context of the TALASUR study.

In case of complete response under study drugs, the combined treatment will be stopped after 2 years after the documentation of this complete response.

Tumor evaluation (thoracic and abdomino-pelvic) will be performed on CT-scan or MRI machine at baseline, every 8 weeks, at the end of study treatment, and thereafter every 8 weeks in the absence of tumoral progression. Disease assessment evaluation will be determined locally by the investigator according to RECIST criteria 1.1.

A Quality of life assessment (EORTC QLQ-C30 and EQ-5D self-administered questionnaires) will be performed at baseline, every 8 weeks and at the end of treatment.

### Statistical design overview

The TALASUR study is a multicenter single-arm phase II trial.

#### Sample size calculation

Based on JAVELIN Bladder 100 results (PFS of 3.4 months in Avelumab arm), the following hypotheses were chosen: a median PFS of 4 months will be considered as an unacceptable efficacy endpoint whereas a median PFS of 7 months will be considered as an acceptable endpoint showing efficacy of this combination. Assuming a 5% (one tailed) type I error, according to a one-arm survival single stage design, 45 assessable patients will be required in the trial to ensure a 80% power (SWOG Cancer Research and Biostatistics). The median PFS should be longer than 5.9 months for the study to be positive. To anticipate 10% of non-assessable patients, we plan to enrol 50 patients overall over 18 months of inclusion.

#### Statistical analyses

All outcomes will be analysed based on intention-to-treat principle. PFS, OS and local/distant disease control rates will be estimated via the Kaplan–Meier method and median follow-up will be calculated using the reverse Kaplan–Meier method, from which the median and 95% CI will be calculated. Patients who will die without reported prior progression will be considered to have progressed on the day of their death. Patients without documented death at the time of the final analysis will be censored at the date of the last follow-up that verified absence of progression. As for the exploratory analyses, we will consider using proportional Cox regression to evaluate the association between time-to-event outcomes and covariates such as PD-L1 status.

Adverse events during the study will be tabulated overall and further tabulation will be made based on time of occurrence and relationship to treatment. The latter excludes events unrelated or not likely related to treatment, but includes events for which the relationship with treatment is not assessable. Tolerability will be summarized by duration of treatment, reasons of discontinuation, dose reduction rates and reasons for dose reductions.

Quality of life (QoL) scores and changes from baseline scores will be described for selected primary scales. Missing values will be calculated such that if at least half the items from the scale will be completed, it will be assumed that the missing items will have values equal to the average of those items present. The Z-test or the non-parametric Wilcoxon–Mann–Whitney tests will be used to evaluate the evolution in global health status and other dimensions of the EORTC QLQ-C30 and EQ-5D. Survival without deterioration in QoL will be defined as the time interval between the date of initiation of Talazoparib plus Avelumab treatment and date of the first deterioration. Deterioration from baseline is defined as a “minimal clinically important difference” (MCID) of > 10-point out of 100. Deterioration will be considered in at least one of the following dimensions: global health-related QoL, and fatigue, without significant clinical improvement subsequently or death, regardless of cause. Survival without deterioration in QoL will be estimated with the Q-TWIST (Quality-Adjusted Time Without Symptoms or Toxicity) method.and.

All the analyses will be performed in R (Version 4.0.2) and will be two sided with p value less than 0.05 considered as significant.

#### Independent data monitoring committee

An Independent Data Monitoring Committee (IDMC) will be set-up to ensure the protection of patients, to ensure the ethical conduct of the study, to evaluate the benefit/risk ratio of the study and to insure an independent review of the scientific outcomes during and at completion of the study.

The committee will include a biostatistician, a pharmacologist and a medical oncologist.

The members of the IDMC will be consulted at least before the trial initiation, and at the final analysis.

#### Data management

A Web Based Data Capture (WBDC) system will be used for data collection and query handling. The investigator will ensure that data are recorded on the eCRFs as specified in the study protocol and in accordance with the instructions provided.

The investigator ensures the accuracy, completeness, and timeliness o

f the data recorded and of the provision of answers to data queries according to the Clinical Study Agreement. The investigator will sign the completed eCRFs. A copy of the completed eCRFs will be archived at the study site.

### Treatment discontinuation and withdrawal from study

The reasons for why a patient may discontinue to participate to the study or interrupt study treatment include the following circumstances.

#### Treatment discontinuation

Avelumab and/or Talazoparib will be administered until the investigator considers that the patient no longer obtains benefit from it. Avelumab and/or Talazoparib will be interrupted at any time under the following circumstances:

- Unacceptable toxicity requiring discontinuation of both treatments.
- A period of 8 weeks with no doses of Avelumab and/or Talazoparib is deemed to be treatment discontinuation and withdrawal of the patient from the study.
- Disease progression
- Need to initiate another systemic anti-tumor treatment
- Patient’s decision (the data already collected during the search can be kept and exploited unless the patient opposes it)
- Patient withdrawal
- Investigator’s decision
- Concomitant illness or other reason that necessitates stopping treatment of the study
- Protocol violation
- Patient lost to follow-up
- Death
- Study terminated by Sponsor

In the event of study termination due to disease progression, non-response to treatment, or early termination of the study for any cause, management of patient including subsequent anti-tumor treatment will be at the investigator’s discretion.

#### Withdrawal from study

Reasons a patient may discontinue treatment include, but are not limited to:

- Treatment failure/confirmed disease progression
- Major protocol violation
- Intolerable toxicity
- Concomitant disease or other reason requiring the discontinuation of treatment
- Patient request (withdrawal of consent for further treatment)
- Investigator’s request (with detailed documentation of reasoning)
- Non-compliance of patient
- Trial termination by the sponsor
- Pregnancy
- Death

## Discussion

The recommended first line treatment of mUC is a platinum-based chemotherapy. This treatment is efficient with an ORR higher than > 50%. However, the PFS remains short (around 7.7 months) and the chemotherapy is too toxic to be used in a prolonged time. Traditionally, maintenance systemic therapy refers to the utilization of regimens with less toxicity given to patients whose cancer is controlled after the initial upfront treatment. The aim of maintenance treatment is to prolong the disease control obtained with initial treatment. This concept has already been shown with PARPi in ovarian carcinoma, as well as, more recently, with Durvalumab in lung carcinoma and Avelumab in UC which is now the standard of care of maintenance treatment after response or stability to the first line platinum treatment.

The prevalence of somatic mutations in HR genes in UC, as well as the known relationship between DDR deficiency and platinum sensitivity, and the recent results from the Atlantis trial with rucaparib treatment in DDR tumors suggest Talazoparib, a PARPi, to be a target for a maintenance treatment of UC. Moreover, there is a strong rationale with both pre-clinical and clinical data to associate Avelumab and Talazoparib.

In addition to the assessment of the clinical outcomes of Avelumab plus Talazoparib in mUC in maintenance therapy after platinum-based chemotherapy, the translational explorations will shed light on some potential determinants of the efficacy of the dual treatment. UC is indeed known to be a heterogeneous disease characterized by genomic instability and a high mutation rate. According to the recent findings from the exploratory biomarkers analysis of the Javelin 100 trial, OS appeared to be modestly associated with mutations in individual genes such as BRCA1 and BRCA2. However, considering molecular signatures, an OS benefit in PD-L1 positive subgroup and tumors with mutated DDR genes was observed, reinforcing the relevance of investigating the molecular markers that may impact on the benefit of a maintenance treatment combining Avelumab and Talazoparib [23].

## Conclusion

Findings from recent phase II trials showed some clinical benefits of PARPi treatment in UC, but the tumor biomarker selection and the treatment indication remain debated. The single arm phase II TALASUR trial will evaluate the association of Avelumab and Talazoparib in maintenance setting in platinum sensible and unselected biomarker mUC to determine the efficacy and the safety of the combination in this indication and investigate predictive biomarkers in explorative ancillary studies.

## Data Availability

Not applicable.

## Abbreviations

BSC: Best supportive care
CR: Complete response
CT: Computed Tomography
CTCAE: Common Terminology Criteria for Adverse Events
DCIS: Ductal carcinoma in situ
ECOG: Eastern Cooperative Oncology Group
FDA: Food and Drug Administration
FFPE: Formalin-fixed, paraffin-embedded
GETUG: Groupe d’Etude des Tumeurs Uro-Génitales
ICI: Immune checkpoint inhibitor
IDMC: Independent Data Monitoring Committee
MRI: Magnetic Resonance Imaging
NCI: National Cancer Institute
ORR: Objective response rate
OS: Overall survival
PARPi: Poly(ADP-ribose) polymerase inhibitor
PD 1: Programmed death 1
PDL 1: Programmed death ligand 1
PFS: Progression-free survival
PR: Partial response
QoL: Quality of life
Q-TWIST: Quality-Adjusted Time Without Symptoms or Toxicity
RECIST: Response Evaluation Criteria in Solid Tumors
RR: Response rate
SPIRIT: Standard Protocol Items, Recommendations for Interventional Trials
TST: Time to subsequent therapy
UC: Urothelial carcinoma
